# *In Vivo* and *In Vitro* Genome-Wide Profiling of RNA Secondary Structures Reveals Key Regulatory Features in *Plasmodium falciparum*

**DOI:** 10.3389/fcimb.2021.673966

**Published:** 2021-05-17

**Authors:** Yanwei Qi, Yuhong Zhang, Guixing Zheng, Bingxia Chen, Mengxin Zhang, Jian Li, Tao Peng, Jun Huang, Xinhua Wang

**Affiliations:** ^1^ Department of Pathogenic Biology and Immunology, School of Basic Medical Sciences, Guangzhou Medical University, Guangzhou, China; ^2^ Department of Blood Transfusion, The First Affiliated Hospital of Guangzhou Medical University, Guangzhou Medical University, Guangzhou, China; ^3^ The Third Clinical School, Guangzhou Medical University, Guangzhou, China; ^4^ State Key Laboratory of Cellular Stress Biology, Innovation Center for Cell Signaling Network, School of Life Sciences, Xiamen University, Xiamen, China; ^5^ Sino-French Hoffmann Institute, State Key Laboratory of Respiratory Disease, School of Basic Medical Science, Guangzhou Medical University, Guangzhou, China; ^6^ The First Affiliated Hospital of Guangzhou Medical University, Guangzhou Medical University, Guangzhou, China

**Keywords:** *Plasmodium falciparum*, RNA secondary structure, *in vivo*, *in vitro*, mRNA

## Abstract

It is widely accepted that the structure of RNA plays important roles in a number of biological processes, such as polyadenylation, splicing, and catalytic functions. Dynamic changes in RNA structure are able to regulate the gene expression programme and can be used as a highly specific and subtle mechanism for governing cellular processes. However, the nature of most RNA secondary structures in *Plasmodium falciparum* has not been determined. To investigate the genome-wide RNA secondary structural features at single-nucleotide resolution in *P. falciparum*, we applied a novel high-throughput method utilizing the chemical modification of RNA structures to characterize these structures. Structural data from parasites are in close agreement with the known 18S ribosomal RNA secondary structures of *P. falciparum* and can help to predict the *in vivo* RNA secondary structure of a total of 3,396 transcripts in the ring-stage and trophozoite-stage developmental cycles. By parallel analysis of RNA structures *in vivo* and *in vitro* during the *Plasmodium* parasite ring-stage and trophozoite-stage intraerythrocytic developmental cycles, we identified some key regulatory features. Recent studies have established that the RNA structure is a ubiquitous and fundamental regulator of gene expression. Our study indicate that there is a critical connection between RNA secondary structure and mRNA abundance during the complex biological programme of *P. falciparum*. This work presents a useful framework and important results, which may facilitate further research investigating the interactions between RNA secondary structure and the complex biological programme in *P. falciparum*. The RNA secondary structure characterized in this study has potential applications and important implications regarding the identification of RNA structural elements, which are important for parasite infection and elucidating host-parasite interactions and parasites in the environment.

## Introduction

RNA, a multitasking biomolecule, plays important roles in many aspects of cellular and physiological processes, such as the regulation of transcription, RNA processing and stability including splicing events, and translation (Wan et al., 2011; Mortimer et al., 2014). As the carrier of genetic information for translation into proteins, RNA molecules can fold into a wide array of complicated and exquisite secondary and tertiary structures *via* complex patterns of intramolecular base pairing formed by Watson-Crick-base pairing (Vandivier et al., 2016). These complex secondary and tertiary structures of RNAs play important roles in regulating such processes as catalytic and ligand sensing, the regulation of mRNA maturation, translation and turnover, alternative polyadenylation and alternative splicing (Ding et al., 2014).

Due to the multiple functions of RNA structural elements changed by base pairing and folding, they can be considered another layer of the genetic code that is only beginning to be understood (Bevilacqua et al., 2016). Characterizing these complex global RNA structures, especially *in vivo*, is essential to achieve a mechanistic understanding of the function and regulation of RNA transcripts. However, it remains challenging to understand the role and mechanism of mRNA secondary structure-based regulation, especially the long-standing challenges in RNA structure modelling. Although structural studies can examine global patterns of RNA structures in a single sample, these RNA structures are not static *in vivo*. At the same time, RNA structure can be refolded by RNA binding proteins or have posttranscriptional covalent modifications that result in drastic changes in secondary structure in response to changes in the prevailing physicochemical environment of the cell and the effects of various stimuli (Vandivier et al., 2016). These studies may help to identify additional temperature sensors (RNA thermometers), enzymes (ribozymes), ligand-binding sensors (riboswitches) or other environmentally responsive structural elements in various organisms, although not in *Plasmodium* parasites.

Investigation of RNA secondary structures associated with specific biological events is therefore essential to understanding the functions and roles of these RNA molecules. The secondary structure of RNA is relatively stable and is present across the length of an mRNA, including the CDS and UTRs (Mignone et al., 2002). A number of studies using global RNA structure-probing approaches have explored the RNA structurome in different species, such as mouse cell lines (Spitale et al., 2015), yeast (Kertesz, 2010; Talkish et al., 2014), human cells (Wan et al., 2014), *Arabidopsis thaliana* (Zheng et al., 2010; Li et al., 2012b; Ding et al., 2014; Incarnato et al., 2014), *Drosophila melanogaster* and *Caenorhabditis elegans* (Li et al., 2012a), zebrafish (Kaushik et al., 2018), *Yersinia pseudotuberculosis* (Righetti et al., 2016), and, recently *Oryza sativa* (Su et al., 2018). However, a comprehensive whole-genome analysis of RNA secondary structures has not been obtained for *Plasmodium* parasites. Furthermore, a thorough analysis of secondary structure correlations between different developmental stages has never been accomplished. In this study, we applied the novel biochemical approach icSHAPE which, to the best of our knowledge, enables the first global view of RNA secondary structures in the major human pathogen *Plasmodium falciparum* for all four bases *in vivo* and *in vitro* at the ring stage and trophozoite stage of parasite development.

Malaria is caused by any one of five species of protozoan parasites, namely, *P. falciparum*, *P. vivax*, *P. malariae*, *P. ovale*, and *P. knowlesi*, and is one of the most important tropical parasitic diseases with high morbidity and mortality rates, especially in many developing countries. In 2019, approximately 229 millions cases of malaria and 409,000 deaths were reported in endemic countries each year, especially in the sub-Saharan African region (World Health Organization, 2020). Various measures have been employed to control the disease, including vector control, bed-nets, and chemotherapy, but these methods have achieved only limited success (Choi et al., 2019). Given the lack of effective vaccines, the widespread resistance to anti-malaria drugs in current use (Capela et al., 2019), and poorly understood molecular mechanisms, it has become an urgently important to identify and develop new effective strategies to control malaria.

The major strength of this study is that it explained the landscape of the RNA secondary structure associated with parasite development at single-nucleotide resolution. Our analysis profiled the structure of more than 3,396 (*in vivo*) and 2,024 (*in vitro*) transcripts in the ring-stage and trophozoite-stage developmental cycles. In this study, we showed that (1) RNA secondary structures in *P. falciparum* have common characteristics similar to those of other diverse organisms, such as UTR regions, CDS regions, noncoding RNAs, and malaria mitochondrion. (2) Structural changes are dramatically different in the ring and trophozoite stages but less pronounced in the ring stage than in the trophozoite stage. (3) Comparing the *in vivo* and *in vitro* structures indicates the important role of RNA-binding protein (RBP) in structure formation. (4) Combined with transcriptome data, RNA secondary structure changes were significantly associated with transcriptome changes. Therefore, our results make it possible to obtain a framework for understanding how malaria parasites develop through changes in RNA structure *in vivo* and potential use for further investigations. Our results imply that there is a critical connection between the RNA secondary structure and the complex biological programme of *Plasmodium falciparum*, but the mechanism governing this process needs to be further elucidated.

## Materials and Methods

### Parasite Culture and Synchronized

The *P. falciparum* strain 3D7 was cultured in human O^+^ erythrocytes at 5% hematocrit under standard *in vitro* conditions as previously described (Trager and Jensen, 1976). Cultures were synchronized twice at ring stage with 5% D-sorbitol treatments performed 8 hours apart (Lambros and Vanderberg, 1979). The cultures were expanded to accommodate harvesting of at least 200 mL of culture at each planned time-point. Cultures (~8% parasitemia in 5% hematocrit) were harvested 48 hours (ring stage) and 72 hours (trophozoite stage) after the first sorbitol treatment. Next, the cells were harvested at 2000 rpm for 3 min to obtain packed RBCs. The packed RBCs were lysed by a 0.05% saponin solution, and parasite pellets were washed twice using phosphate-buffered saline (pH 7.4). The remaining pellets were used for icSHAPE library construction, and total RNA was extracted directly, or conversely, parasites were stored in 10 mL of TRIzol reagent at −80°C prior to RNA extraction.

### *In Vivo* and *In Vitro* NAI-N_3_ Chemical Probing and RNA Sample Extraction

For *in vivo* NAI-N_3_ modification of parasite RNAs, the washed parasites pellets were treated as described previously (Flynn et al., 2016) before total RNA extraction. The parasites pellets were equally divided into three parts, one part for NAI-N_3_ *in vivo* modification and the two other parts for the DMSO control. NAI-N_3_ concentration/times were chosen as in our experiment described in the following section to provide a similar overall level of modification for samples *in vivo*. Briefly, almost all PBS was removed from the pelleted parasite, the parasite pellet was resuspended in 200 µl of 100 mM NAI-N_3_ solution or 200 µl of DMSO solution, and the suspension was immediately mixed by inversion and incubated at 37°C with end-over-end rotation for 15 min. Before use, the 100 mM NAI-N_3_ was thawed completely to 37°C. The reaction was stopped by quickly placing the pellets on ice and collecting them by centrifugation at 14,000 g at 4°C for 30 s. Next, the supernatants were removed, and the pellets were transferred to 15-ml tubes and resuspended in 10 ml of prewarmed TRIzol Reagent. Total RNA was isolated as recommended by the manufacturer’s protocol followed by phenol/chloroform extraction and ethanol precipitation. The RNA yield was measured using a Nanodrop spectrophotometer, and RNA quality was assessed by 1% agarose gel analysis. We ensured that clear 18S and 28S rRNA bands were present. We needed to construct total RNA/polyA-selected RNA *in vivo* SHAPE RNA libraries. In this study, polyA mRNA was obtained using the NEBNext^®^ Poly-A mRNA Magnetic Isolation Module (NEB).

For *in vitro* NAI-N_3_ probing, we also needed to construct total RNA/ribosomal *in vitro* SHAPE RNA libraries. For total RNA/ribosomal *in vitro* SHAPE RNA libraries, no additional processing was needed. For polyA-selected *in vitro* SHAPE libraries, polyA selection was performed first using polyT oligomagnetic beads before NAI-N_3_ *in vitro* modification was undertaken. Heat-denatured total RNA or polyA+ RNA samples were obtained from DMSO-treated pellets at 95°C for 2 min and were later transferred to ice to cool. The NAI-N_3_ final concentration and times for *in vitro* modification were 100 mM and 37°C for 10 min. The reactions were stopped by moving the samples to ice, and the samples were purified using a Zymo RNA Clean & Concentrator-5 column according to the manufacturer’s protocol.

### Quantification of Modifications by Primer Extension and Resolution by Capillary Electrophoresis

Incubation time and NAI-N_3_ concentration can strongly affect single-hit kinetics; therefore, we focused on optimizing these two parameters, although it is possible that other factors, such as temperature and buffer conditions, could also affect these kinetics (Ding et al., 2015). We took a time course (5 min, 10 min, 15 min and 30 min) of *in vivo* NAI-N_3_ modification as an example for a region of 18S rRNA in *P. falciparum* by capillary electrophoresis for at least three times. Seven micrograms of total RNA (non-polyA-selected) was treated with DNase (NEB) to remove residual genomic DNA in the RNA sample. Next, the DNA-free total RNA was reverse-transcribed using SuperScript III (Invitrogen) by a colour-coded fluorescently FAM-labelled oligonucleotide in the 271-bp-293-bp region of the 18S rRNA gene. The PCR programme was as follows: samples were heated to 65°C for 5 min, maintained on ice for at least 2 min, and incubated at 50°C for 1 h followed by 95°C for 5 min, and the samples were maintained at 4°C after the reaction ended. The reverse-transcribed cDNA products were electrophoresed using an ABI 3730XL DNA Sequencer. The results were presented by GeneMarker (V2.7.0, SoftGenetics, LLC.)

### Determination of Fragmentation Time by Primer Extension Electropherograms

Total RNA was extracted from parasite lysates without any chemical reagent modification. In a 20-μL reaction, ~2 μg of total RNA was fragmented using RNA fragmentation reagents (Ambion) for the proper time at 70°C. After the end of the experiment, 10× stop solution was added to each sample, and the samples were immediately placed on ice. Next, fragmented RNA was purified with a Zymo RNA Clean & Concentrator-5 column. The purified fragmented RNA was reverse-transcribed by primer extension with gene-specific colour-coded fluorescently FAM-labelled oligonucleotides (5’-ACCCTAACATCAAAAGCTGATAGG-3’), as described above. The length of every fragment was assessed using an ABI 3730XL DNA Sequencer (Applied Biosystems). The results were shown by GeneMarker (V2.7.0, SoftGenetics, LLC.).

### RNA-Seq Illumina Library Construction

For RNA-seq library construction, the NEBNext Ultra Directional RNA Library Prep Kit (NEB) was used to prepare a validated strand-specific, polyA-selected RNA sequencing library according to the manufacturer’s instructions in the kit. Briefly, mRNA was purified from 3 µg of total RNA using the Poly(A) mRNA Magnetic Isolation Module. Fragmentation was carried out at 94°C for 7 min in NEBNext First Strand Synthesis Reaction Buffer. First strand cDNA was synthesized using random primers and ProtoScript II Reverse Transcriptase (with 0.1 µg/µl actinomycin D and murine RNase inhibitor). Second strand cDNA was synthesized using Second Strand Synthesis Enzyme Mix and incubated at 16°C for 1 h. Next, the double-stranded cDNA was purified using 1.8X Agencourt AMPure XP Beads. End prep of a cDNA library was performed by NEBNext End Prep Enzyme Mix at 20°C for 30 min followed by 65°C for 30 min. NEBNext adaptors were ligated to the cDNAs with the function of Blunt/TA Ligase Master Mix at 20°C for 15 min, and fragments measuring 150–200 bp were purified using AMPure XP beads. The samples were later treated with 3 μl NEBNext USER Enzyme at 37°C for 15 min before PCR amplification. Library amplification was performed using NEBNext Q5 Hot Start HiFi PCR Master Mix, NEBNext Universal PCR Primer for Illumina, and Index (X) Primer. The primer and oligonucleotide sequences used in the experiment are shown in **Supplementary Table 1**. The PCR machine was programmed as follows: initial denaturation at 98°C for 30 s, denaturation at 98°C for 10 s and annealing/extension at 65°C for 75 s for 12 cycles. The end step was final extension at 65°C for 5 min. The PCR products were purified using an AMPure XP system before the quality of the libraries was assessed using an Agilent Bioanalyzer 2100, and the libraries were sequenced on an Illumina HiSeq 2000 at Vazyme Biotech Co., Ltd. (Nanjing, China) and GENEWIZ company (Suzhou, China). Paired-end reads were mapped to the *P. falciparum* 3D7 assembly release-33 downloaded from PlasmoDB (http://plasmodb.org/common/downloads/Current_Release/Pfalciparum3D7/). Next, read counts were collected, and gene expression levels among different samples were estimated.

### icSHAPE Deep-Sequencing Illumina Library Construction

For the icSHAPE library, we used the methods developed by Ryan A Flynn et al. as described previously (Flynn et al., 2016), with some modifications. For total RNA/ribosomal RNA libraries, no additional processing was needed. For polyA-selected libraries, polyA selection was performed first using polyT oligo magnetic beads. Briefly, DNA-free mRNA, which was purified from total RNA using polyT oligo magnetic beads, was subjected to a biotin click reaction with the modification reagent NAI-N_3_ at 37°C in a thermomixer for 2 h at 1,000 r.p.m. with 4 U/µl RiboLock RNase inhibitor. Fragmentation was performed using RNA fragmentation reagent (Ambion) at 70°C for 3 min. Next, purified fragmented RNA was end-repaired by T4 polynucleotide kinase (NEB) and incubated at 37°C for 1 h. A 3’-biotin RNA linker or a 3’-ddC RNA linker were ligated to the end-repaired RNA samples by T4 RNA ligase 1. After each above step, the reaction was purified by a Zymo RNA Clean & Concentrator-5 column according to the manufacturer’s protocol.

First strand cDNA was subsequently synthesized using 1 µl of 1 µM RT primer (with a 4-nt barcode for separate samples in one sequencing lane). After reverse transcription, isolation by MyOneC1 streptavidin beads and size selection (>70 nt) of NAI-N_3_-modified molecules from cDNA products, the concentrated cDNA samples were purified with a Zymo DNA Clean & Concentrator-5 column. The size-selected cDNA was circularized by 5 U/µl CircLigase II (Epicentre) and incubated at 60°C for 2 h. Amplification was performed using NEBNext High-Fidelity 2X PCR Master Mix, Solexa-P5 primer, and Solexa-P3 primer. The qPCR machine was programmed as follows: 98°C for 45 s, 98°C for 15 s, 65°C for 20 s, and 72°C for 30 s. For the *in vivo* and *in vitro* NAI-N_3_-treated samples, 14 cycles were employed, and for DMSO-treated samples, 11 cycles were employed. Finally, the PCR-amplified library was gel size-selected using 10% TBE PAGE to obtain a size between 200 bp and 300 bp. The primer and oligonucleotide sequences used in the experiment are shown in **Supplementary Table 1**.

Final icSHAPE library material was quantified on a Bioanalyzer High Sensitivity DNA Chip 2100 (Agilent) and Qubit and was sent for deep sequencing and Illumina HiSeq analysis at Vazyme Biotech Co., Ltd. (Nanjing, China) and GENEWIZ Company (Suzhou, China).

### Sequencing Data Analysis

The quality of all PF data from Illumina’s HiSeq sequencer was assessed by FastQC (Babraham Bioinformatics), and the percentages of reads with Q20, Q30, and GC content were calculated. All sequenced libraries were collapsed to remove PCR duplicates, reads containing adapters, reads containing polyN, and low-quality scores.

All downstream analyses were based on clean data with barcodes (1-13 bases in each read) removed by using Trimmomatic (Bolger et al., 2014), and reads with bases < 35 nt were discarded after barcode removal. All the clipped sequenced libraries were later mapped to the *P. falciparum* genome and transcriptome (build release-33 downloaded from PlasmoDB on June 26th, 2017 http://plasmodb.org/common/downloads/Current_Release/Pfalciparum3D7/) using bowtie (Langmead et al., 2009) implemented with mismatch = 0 (bases from 1-28 nt) and default parameters. The ‘-1 positions’ of each sequencing read represented the reverse transcription stop, which corresponded to modified nucleotides in the NAI-N_3_ group and intrinsic/fragmentation modified in the DMSO control group. The number of times a base was mapped as ‘-1 positions’ was counted by Perl scripts and bed tools (Quinlan and Hall, 2010). All icSHAPE profiles of all mapped RNA species were sorted into different files by chromosome for the two experiments at the two developmental stages at the ring stage and trophozoite stage. All reads mapped to the genome and transcriptome from different libraries were normalized by the total number of RT stops in each library [NAI-N_3_ *in vivo*, NAI-N_3_ *in vitro*, and DMSO control] and sequencing depth. The icSHAPE signals/scores for each RNA position were calculated as the ratio of NAI-N_3_/DMSO numbers of modified nucleotides after all reverse transcription stops were normalized by the amount of all reads in each library (Talkish et al., 2014). To reduce the potential overestimation of structural signals of bases with low/zero coverage, a small number 5 was added to the numbers of modified nucleotides, which were normalized by sequencing depth (Wan et al., 2014). The final analysis of the icSHAPE sums was performed with Microsoft Excel 2013 and Student’s t-tests using R.

### Computation of the RNA Secondary Structures With icSHAPE Scores

An increasing number of online web services or software can be used for the prediction of RNA secondary structure (Jin et al., 2011). In our research, colour coding by icSHAPE signalling was performed using online ViennaRNA Web Services (http://rna.tbi.univie.ac.at/) with five different colours. Specifically, the coloured base pair was placed into the known regions of the 18S rRNA secondary structure, and the experimental single/double results were subsequently checked against the known ways of pairing. For the RNA secondary structure profile, all selected secondary structure models were generated using RNAstructure 6.1 12 on a Windows operating system (http://rna.urmc.rochester.edu/RNAstructure.html) with and without hard constraints due to the extracted icSHAPE score profiles of all single RNAs that were calculated (After testing and comparing on the known regions of 18S rRNA, we concluded that in RNAstructure software, the parameter of threshold for force single stranded was 1.5, and the threshold for chemical modification was 1.0, for our icSHAPE scores profiles.) In our approach, the nucleotides that have icSHAPE scores above a specified threshold (1.5) are forced to be single-stranded. Otherwise, the positions are treated as being inaccessible to chemical modification, i.e., double-stranded or crowded by specific protein. We also used the current best parameters (read shape reactivity - pseudo energy constraints with slope = 1.8, intercept =-0.613) for icSHAPE-directed RNA structure prediction. Alternatively, webserver tools were also used at http://rna.urmc.rochester.edu/RNAstructureWeb/. RNA structures were drawn and coloured using StructureEditor.

## Results

### Determination of the Conditions for Single-Hit Kinetics and Fragmentation Time by Primer Extension Electropherograms

To obtain the single-nucleotide resolution and genome-wide RNA secondary structural features in *P. falciparum*, NAI-N_3_ should be titrated to single-hit kinetics in structure probing. In our pilot experiments, we used capillary electrophoresis (CE) (Vasa et al., 2008), which has extended the amenable length to 500 nt, to confirm the NAI-N_3_-labelled 18S 5’ end rRNA structure and compare those structures with the known secondary structure of PF 18S rRNAs (Wong et al., 2014) (**Supplementary Figure 1**). Our results showed that we obtained nearly identical secondary structure for the 5’ end of 18S rRNA as previously reported (**Supplementary Figure 1**). These results show that the 15-min time point is the optimal duration for NAI-N_3_ modification, as it is the longest time point for which single-hit kinetics still occur as revealed by consistency with the known structure. The 30-min time point is too long, as revealed by the significant of loss modified points and the increase of shorter length bands. Next, we used this condition for all the following *in vivo* RNA modifications. IcSHAPE *in vivo* libraries were subsequently prepared using RNA from pellets treated with 100 mM NAI-N_3_ for 15 min, and control pellets not treated with NAI-N_3_ but with DMSO solution.

Appropriate fragment sizes have a critical role in ensuring the highest signal to-noise ratio and are need for efficient obtaining meaningful reads and modification sites *via* RT-PCR and sequencing. Short RNAs (measuring~100 nt) are the most common strategies that have been optimized to achieve single-hit kinetics of chemical modification, which can result in ~10% of all transcriptions being structurally informative (Merino et al., 2005). For this purpose, we first determined the fragmentation time by primer extension electropherogram experiments (**Supplementary Figure 2**). The results showed that the 3-min time point was the optimal fragment time for obtaining ~100 nt short RNAs. The 4-min time point is too long, as revealed by significant loss of ~100 nt short RNAs.

### Comparison of icSHAPE Data to Known RNA Structures

**Figure 1:**
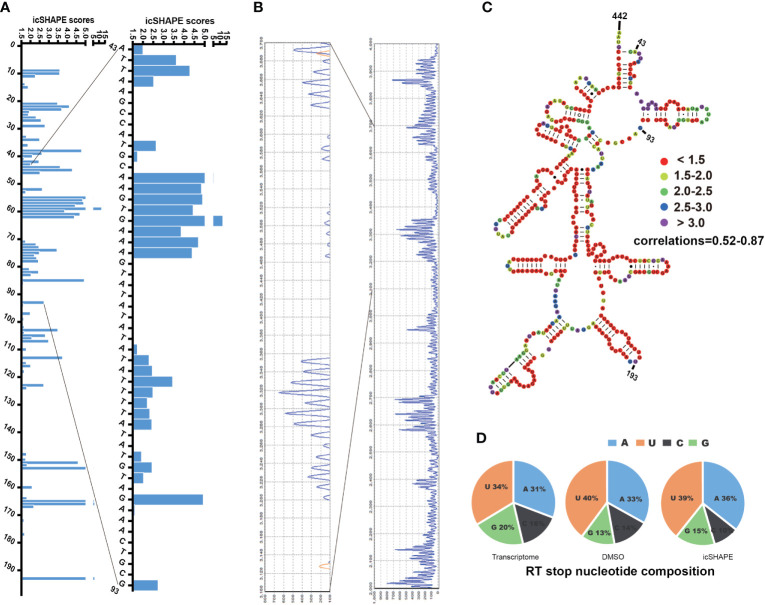
icSHAPE scores accurately maps the region 43–442bp of P. falciparum 18S small subunit rRNA (blood stage) icSHAPE scores accurately maps the region 43–442bp of *Plasmodium falciparum* 18S small subunit ribosomal RNA (blood stage) and agrees with capillary electrophoresis-based *in vivo* structure probing. **(A)** icSHAPE scores for nucleotides 1-193 is shown on the left. The sites of nucleotides which showed have the scores above 1.5, this score means this position is single-strand. **(B)** The gel from 18S A-type rRNA NAI-N_3_ modification read-out by capillary electrophoresis-based probing, which was done here near the 5’ end (100bp-300bp), which consist with the region on the 1-193 of the 18S rRNA. **(C)** Nucleotides 43–442 of the 18S A-type small subunit ribosomal RNA, colour-coded according to icSHAPE scores. The whole secondary structure of 18S A-type small subunit ribosomal RNA are showed in **Supplementary Figure 3**. **(D)** Reverse transcription stop distribution for the transcriptome of 18S A-type rRNA on chromosome 5, DMSO-treated RNA control libraries or icSHAPE-treated RNAs libraries in the same regions of 18S rRNA. All of the three stop distributions are highly concordant, icSHAPE *in vivo* treatment no enriches for any sequencing reads mapping to four bases compared to untreated control, except a slight enrichment in NAI-N_3_ library for As and Us.

To evaluate the accuracy of our NAI-N_3_-labelledicSHAPE approach, we first used capillary electrophoresis-based probing to compare NAI-N_3_ modifications from icSHAPE with those from conventional gel-based modification read-outs. These results indicated strong agreement for the special regions of 18S rRNA tested (**Figures 1A, B**). Second, we mapped our icSHAPE scores to well-studied RNA molecules (**Figure 1C** and **Supplementary Figure 3**). The secondary structure of *P. falciparum* A-type 18S small subunit ribosomal RNA (http://www.rna.ccbb.utexas.edu) has been published (Cannone et al., 2002); therefore, we used this known RNA secondary structure as a model to compare with our icSHAPE modification RNA secondary structure according to our icSHAPE scores. *In vitro* RNA icSHAPE libraries prepared using RNA from *in vitro* NAI-N_3_ modification with no ribosomal removal were sequenced to produce an average of 14.5 million reads in the 2092-bp region of *P. falciparum* A-type 18S small subunit ribosomal RNA. The icSHAPE scores for each site on 18S rRNA are depicted in **Supplementary Table 2**. Colour coding by icSHAPE signal was performed using online ViennaRNA Web Services (http://rna.tbi.univie.ac.at/) with five different colours according to the icSHAPE scores generated from icSHAPE-seq (icSHAPE scores < 1.5 were marked in red; icSHAPE scores 1.5-2.0 were marked in yellow; icSHAPE scores 2.0-2.5 were marked in green; icSHAPE scores 2.5-3.0 were marked in blue; icSHAPE scores > 3.0 were marked in purple). The coloured base pair was placed into the known regions of the 18S rRNA secondary structure, and the experimental single/double results were subsequently checked against the known ways of pairing.

In the known secondary RNA structure of entire 18S A-type rRNA (length of 2,092 nt, located on chromosome 5), approximately 41.0% of bases are single-stranded, 55.2% of bases are double-stranded, and 3.8% (80 nt) are indeterminate. In the entire 18S A-type rRNA, 43.9% (the icSHAPE score is above 1.5, indicating that this position is single-stranded) of the bases that show high *in vivo* icSHAPE scores (defined as 1.5) in our data set correspond to single-stranded regions in the phylogenetic structure (41.0%), whereas 56.1% (the icSHAPE score is below 1.5, indicating that this position is a double strand) of the bases that show low *in vivo* icSHAPE scores in our dataset correspond to base-paired regions in the phylogenetic structure (55.2%) and presumably are protected by either ribosomal proteins or non-canonical base-pairing tertiary RNA structures. Our data showed that there is a high correlation between icSHAPE scores and the known 18S A-type RNA secondary structure. We set the sites with icSHAPE scores above 1.5 to be localized to regions of single-stranded RNA and were consistent with traditional primer extension analysis and known RNA secondary structures. NAI-N_3_ modification has no specific RT stops with almost equal numbers of four bases, consistent with the known absent or minor (Spitale et al., 2015) (with a very slight enrichment in NAI-N_3_ samples for As and Us) base-specificity of NAI-N_3_ (**Figure 1D**). Overall, the reactivities from our icSHAPE libraries are consistent with the known structural mapping of 18S rRNA, which is the closest *in vivo* model.

### Overview of the *P. falciparum* RNA Structurome

To obtain a genome-wide profile of the single-nucleotide resolution RNA secondary structure during the intraerythrocytic developmental cycle of *P. falciparum*, we performed icSHAPE, as depicted in **Supplementary Figure 4**. *P. falciparum* 3D7 synchronized twice with 5% D-sorbitol was cultured in a 37°C incubator to keep the majority of the parasite population at the same stage. Smears were made by Giemsa staining and used for microscopic observation of parasite morphology at each time point.

The transcripts, with reverse transcription stop coverage no less than 2 and background base density higher than 200 as described previously (Spitale et al., 2015), were analysed. The detailed data in the icSHAPE libraries and RNA-seq libraries are displayed in **Supplementary Table 3**. We obtained an average of 55.38% mappability and 78.3 million qualified fragments per sample in the icSHAPE library (**Supplementary Table 3**). The results are highly reproducible across two biological replicates.

**Figure 2:**
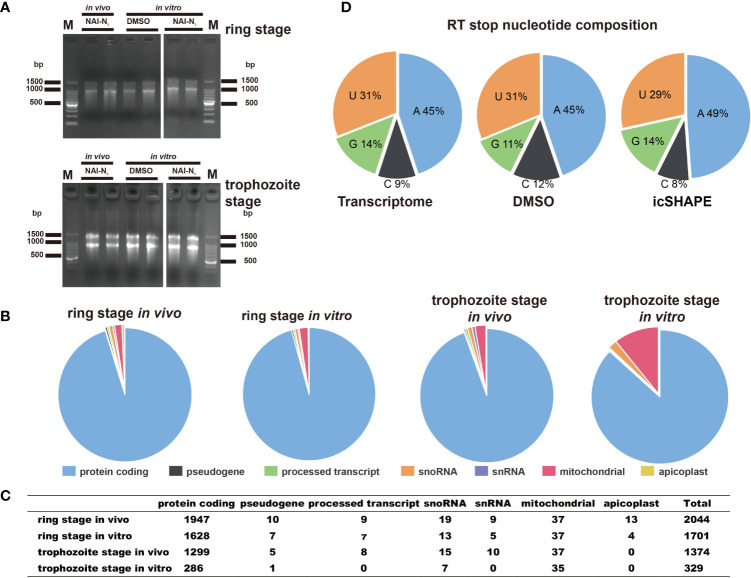
icSHAPE is capable of measuring the RNA structure profiles of thousands of RNAs simultaneously *in vivo* and *in vitro*. icSHAPE is capable of measuring the RNA structure profiles of thousands of RNAs simultaneously *in vivo*. **(A)** Evaluation of RNA quality of two biological replicates between stages and conditions *in vivo* modification, *in vitro* modification, and DMSO control samples in agarose gel. **(B, C)** The total number of structural profiles for transcripts was classified into different classes of RNAs from a total number of 2044 transcripts for the ring stage library at 37 degree *in vivo*, 1701 transcripts for the ring stage library at 37 degree *in vitro*, 1374 transcripts for the trophozoite stage library at 37 degree *in vivo*, and 329 transcripts for the trophozoite stage library at 37 degree *in vitro*. This figure combines results from both two biological replicates. **(D)** Reverse transcription stop distribution for the whole transcriptome, DMSO-treated RNA control libraries or icSHAPE-treated RNAs libraries. All of the three stop distributions are highly concordant, icSHAPE *in vivo* treatment no enriches for any sequencing reads mapping to four bases compared to untreated control, except a slight enrichment in NAI-N_3_ library for As.

Finally, 2,044 and 1,701 valid structural profiles for transcripts (35.2% and 29.4% of the ratio of all transcripts respectively) were obtained from *in vivo* and *in vitro* 37°C treated polyA-selected RNA ring stage libraries, among which the majority were mRNAs as shown in **Figures 2A–C** (and also in **Supplementary Table 4**). In addition 1,374 and 329 valid structural profiles for transcripts (23.7% and 5.69% of the ratio of all transcripts respectively, with reverse transcription stop coverage no less than 2) were obtained from *in vivo* and *in vitro* 37°C treated polyA-selected RNA trophozoite stage libraries (**Supplementary Table 4**). All of the three reverse transcription stop distributions for the whole transcriptome, DMSO-treated RNA control libraries or icSHAPE-treated RNAs libraries were highly concordant. The icSHAPE *in vivo* treatment did not enrich any sequencing reads mapping to four bases compared to the untreated control, except for a slight enrichment in the NAI-N_3_ library for As (**Figure 2D**).

While polyA purification was undertaken in those polyA-selected RNA libraries before library construction, it strongly reduced the reads mapped to the tRNAs and rRNAs. We mapped the structural profiles by icSHAPE score data *in vivo* and *in vitro* from selected regions of 110 mRNAs (the top 110 structural profiles for transcripts that have the highest reverse transcription stop coverage, **Supplementary Table 5**) across parasites that have 5’ untranslated regions (UTRs) and 3’ UTR regions longer than 200 nt: 5’ UTR (200 nt upstream of the start codon); coding sequence region (CDS region, 110 nt downstream of the start codon and 110 nt upstream of the stop codon); and 3’ UTR (100 nt downstream of the stop codon) (Kertesz, 2010). Next, we used the obtained structural profiles by icSHAPE scores to investigate three global properties of parasite transcripts to evaluate the hypothesis that robustness of the periodic structure signal might influence translation and alternative splicing.

### Distribution of icSHAPE Reactivity Profiles *In Vivo* and *In Vitro* Across the CDS Regions and UTR Regions

**Figure 3:**
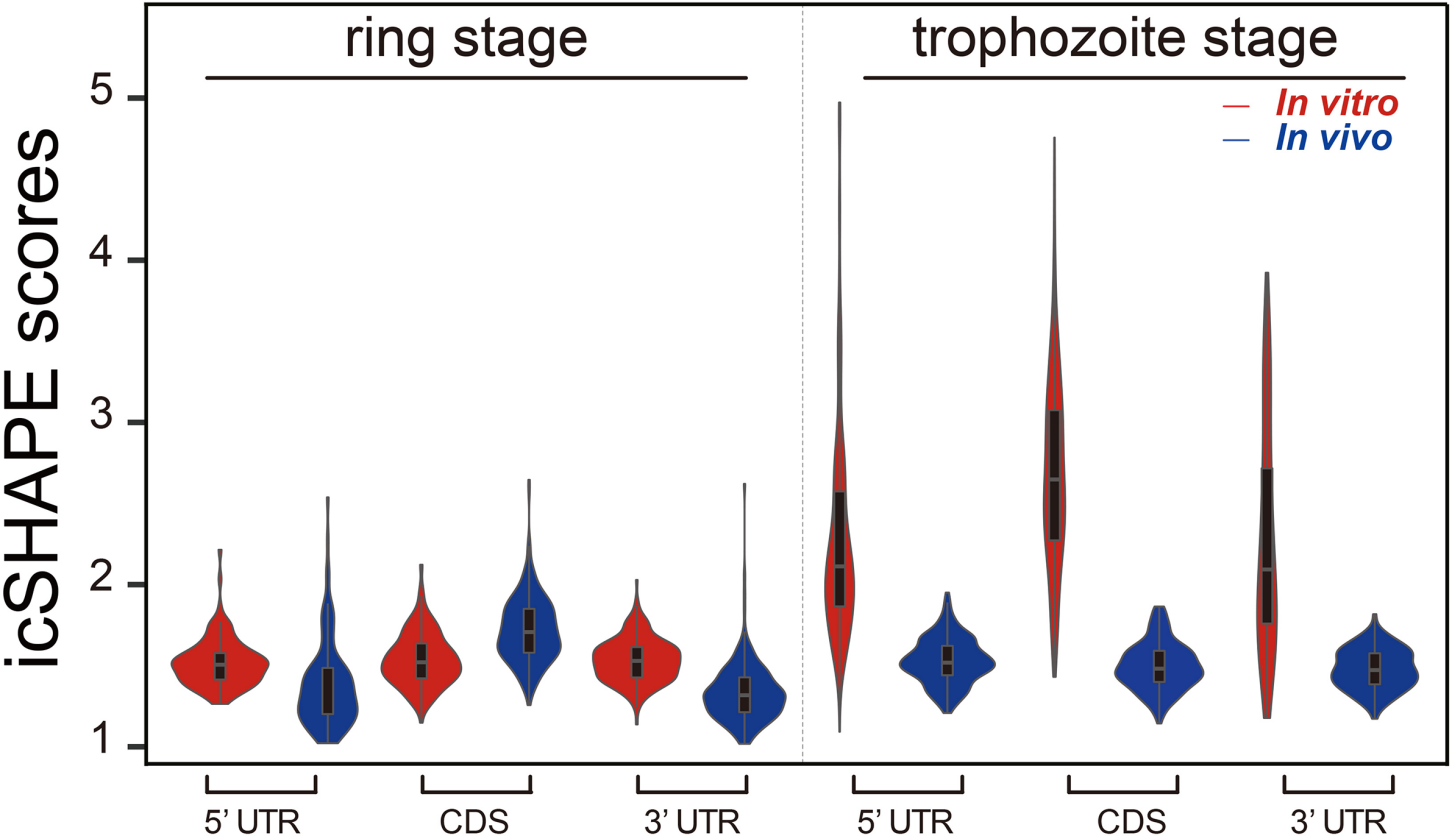
icSHAPE reveals new features of mRNA secondary structures that located 5-UTR, CDS region and 3-UTR region *in vivo* and *in vitro* icSHAPE reveals new features of mRNA secondary structures that located 5-UTR, CDS region and 3-UTR region. The average icSHAPE score *in vivo* and *in vitro* across the CDS regions and UTR regions to quantify the distribution of icSHAPE reactivity profiles in the trophozoite stage development. The figure showed that RNAs are less folded *in vivo* (low icSHAPE scores), and the extent of unfolding varies in different regions of RNAs. The 5’ UTRs exhibited noticeable but partial unfolding, with the largest variation compared to the CDS regions and 3’ UTRs. Those three different regions of RNAs have nearly the same icSHAPE scores *in vivo*, but the CDS region have the highest icSHAPE scores than the 5’ UTR and 3’ UTR when the RNA structure were modified *in vitro*.

The average icSHAPE score of the transcripts *in vivo* and *in vitro* across the CDS regions and UTR regions to quantify the distribution of icSHAPE reactivity profiles in the ring and trophozoite stage development. **Figure 3** shows that RNAs are more folded or crowded by a specific protein *in vivo* (low icSHAPE scores), and the extent of folding varies in different regions of RNAs in ring stage and trophozoite stage. In the ring stage, the 5’ UTRs exhibited noticeable but partial unfolding, with the largest variation being observed between the CDS regions and the 3’ UTRs. These three different regions of RNAs have nearly the same icSHAPE scores *in vivo*, but the CDS region has higher icSHAPE scores than the 5’ UTR and 3’ UTR when the RNA structure is modified *in vitro*. Then, we compared the average icSHAPE score collected from three different regions of RNAs in trophozoite stage, the results also exhibited higher icSHAPE scores *in vitro* than that *in vivo*. Collectively, our data suggest that RNAs are more folded or crowded by a specific protein *in vivo*.

**Figure 4:**
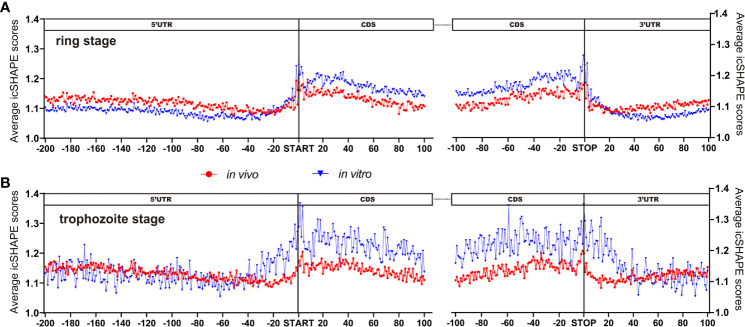
icSHAPE reveals new features of mRNA secondary structures that located 5-UTR, CDS region and 3-UTR region. (Ring stage and trophozoite stage *in vivo* and *in vitro* compare) Average icSHAPE scores in selected regions of the whole expressed transcripts across the ring stage (**A** for ring stage and **B** for trophozoite stage) of parasites that have 5’ and 3’ UTR regions longer than 200 nt: 5’ UTR region (200 nt upstream of the start codon); CDS initial region (100 nt downstream of the start codon); CDS final region (100 nt upstream of the stop codon); and 3’ UTR region (100 nt downstream of the stop codon) are displayed. The start and stop codons of mRNA transcripts are indicated by grey bars; The first 4 nt immediately upstream of the start codon show significantly higher reactivity than the average icSHAPE scores across the first 100 nt of the CDS with Significantly different (Student’s t-test, P < 0.05).

We examined the average icSHAPE score *in vivo* and *in vitro* across the CDS regions and UTR regions to quantify the distribution of icSHAPE reactivity profiles. Due to some nucleotides that were crowded *in vivo* and/or protein binding, or experimental limitations, we observed that icSHAPE scores from *in vivo* and *in vitro* are different. In our research, we found that RNAs are more folded or crowded *in vivo* (low icSHAPE scores), consistent with previous reports (Rouskin et al., 2014; Spitale et al., 2015), and the extent of unfolding was determined to vary in different regions of RNAs (**Figures 4A, B**). It has been revealed that the scale and distribution of RNA structural dynamics under *in vitro* conditions, which are refolded entirely by genome sequencing, are different from those under *in vivo* conditions, in which RNA molecular folding occurs depending on the context of the intracellular environment (Schroeder et al., 2002). Similar results were observed in metazoans (Li et al., 2012a) and humans (Wan et al., 2014), in which UTRs are, on average, more structured than coding regions. However, this global trend is different than that in yeast (Kertesz, 2010) and in *E. coli* (Del Campo et al., 2015), in which UTRs are less structured than CDSs.

### Structure Analysis Across the CDS Regions and UTR Regions *In Vivo*

We performed structure analysis across the CDS regions and UTR regions *in vivo*. By PARS technology, Yue Wan et al. (Wan et al., 2014) were used to analyse more than 3,000 secondary structure human messenger RNAs, and found that it is more complicated and complex RNA secondary structures in the UTR regions than the CDS regions. Structures from the UTR, especially the 5’ UTR, have an important role in the process of development due to their interaction with regulatory proteins. To explore this hypothesis in our experiment, we focused on two structured 5’ UTRs and on the structured 3’ UTR for more detailed functional analyses (**Figures 4A, B**). **Figure 4** shows the icSHAPE signal *in vivo* from the ring stage and trophozoite stage libraries. We compared the average icSHAPE score collected from the four genomic features. The results exhibited significant differences from one region to another region in the RNA structure.

Metagene analysis of the transcripts, which aligned at their start and stop codons, shows that *P. falciparum* CDS regions have a propensity to form double-stranded structure (lower icSHAPE scores) to a level that is similar to the structural propensity of the 5’- and 3’-untranslated regions (UTRs). Additionally, our data indicated that lower icSHAPE scores in the sites from nt -25 to -21 in trophozoite stage than in the other sites of 5’UTR region of the transcriptome. This low icSHAPE scores means that those regions are more structured or protein binding sites of mRNA secondary structures. These positions may provide candidate sites for the functional conformation of mRNA, but require further investigation.

We next investigated whether a correlation is shared between intrinsic mRNA secondary structure propensity around the translation start/stop site and whether the efficiency of protein translation correlates with mRNA abundance. In **Figures 4A, B**, we also found that nt -1 to 4 and the last four base pairs of the CDS region of the transcriptome had significantly (Student’s t-test, P < 0.05) higher icSHAPE scores than average, consistent with previous findings (Ding et al., 2014; Righetti et al., 2016). Notably, the start and stop codons of each transcript exhibit higher icSHAPE scores, indicating reduced tendency for double-stranded conformation and easily increased accessibility for the expression of transcripts.

### mRNA Structure Correlates With mRNA Abundance in *P. falciparum*

It is clear that mRNA structure is important for a variety of biological processes, including maintenance of RNA half-life and stability (Carrier and Keasling, 1997). We next asked whether the RNA secondary structure of the CDS correlates with mRNA abundance in the parasites. To quantify the transcriptome, we performed RNA-seq (**Supplementary Table 6**) with the parasites sample under the same condition as those used for treatment with NAI-N_3_ which exhibited high reproducibility between biological replicates.

**Figure 5:**
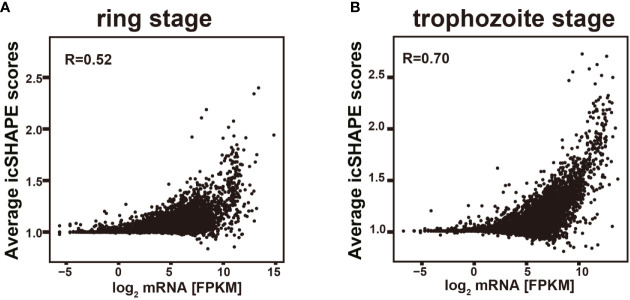
Relationship of icSHAPE and expression abundant (FPKM) from RNA-seq analysis. Comparison of the average icSHAPE scores over the whole transcripts CDS regions revealed a clear correlation with the mRNA abundance. Dependence of the average icSHAPE scores on the mRNA abundance of the whole transcripts from ring stage at 37 degree **(A)**, and also the trophozoite stage at 37 degree **(B)**. Pearson correlation coefficient (R) is showed in each panel.

Comparison of the average icSHAPE score over the CDS revealed a very clear correlation with the mRNA abundance with this combined approach (**Figure 5**). A higher icSHAPE scores, those positions are treated as that they were more accessible to chemical modification, means that those regions are in the presence of a single strand. A lower icSHAPE scores, the positions are treated as that they were unaccessible to chemical modification, indicate the presence of a double strand or those regions are crowded by a specific protein. Our data in **Figure 5** showed that the most abundant transcripts exhibited higher icSHAPE scores. Collectively, our data indicate that it’s has unfolding of structured in those most abundant transcripts and open their double-strand with reduced tendency for double-stranded conformation in order to increase the accessibility for the following of ribosomal protein binding easily. In the other hand, due to some nucleotides which were crowded *in vivo* and/or protein binding, they were not chemically reactive, and no complete coverage was be provided in our experiment for this long transcript, the Pearson correlations in our results seem not high but with reasonable. Therefore, we hypothesized that the RNA structure could regulate the activation/repression of gene expression in *P. falciparum*. Combined analysis of icSHAPE with transcriptome data demonstrated that stage-specific RNA structure plays an important role in the regulation of gene expression in *P. falciparum*.

## Discussion

In this study, to the best of our knowledge, we presented for the first global view of the genome-scale landscape of RNA secondary structures of the major human pathogen *P. falciparum* for all four bases *in vivo* and *in vitro* at two developmental stages at single-nucleotide resolution. The major strength of this scarcity of our RNA secondary structure was its potential use and important implications for understanding parasite gene expression and development. Recent studies have established RNA structure as a ubiquitous and fundamental regulator of gene expression (Mustoe et al., 2018) that can be used as an extremely specific and subtle mechanism for fine tuning a variety of cellular processes (Vandivier et al., 2016) within picoseconds to seconds (Ganser et al., 2019). On the other hand, *Plasmodium* parasites have 4 to 8 nucleus-encoded, structurally distinct, and differentially transcribed rRNA genes in different developmental stages (Gunderson et al., 1987; Qi et al., 2015). This finding also prompted us to identify the RNA secondary structure as an important factor that contributes to posttranslational control, especially in *Plasmodium* parasites.

Prior to NAI-N_3_ treatment, NAI-N_3_ was titred to single-hit kinetics in structure probing at the different temperatures to ensure that on average, any one copy of any RNA molecule was methylated only once. The major difference between the *in vivo* and *in vitro* conditions for SHAPE mapping is the cell walls and membranes encasing the transcriptomes of these organisms (Lee et al., 2017), especially the parasites that are located in the RBCs. For mapping RNA structures using either nuclease (RNase S1/RNase V1) or DMS, NAI-N_3_ chemical probes, single-hit kinetics are one of the key factors (Kwok et al., 2015), because the over/no modification and/or cleavage of each site can cause conformational changes that lead to erroneous conclusions for specific structures. Moreover, the cell walls and cell membranes encasing the transcriptomes of different organisms are different, which makes the single-hit kinetics for diverse organisms different. Finally, we observed that modifying RNA inside parasite cells with 100 mM NAI-N_3_ for 15 min yielded a good-quality RNA secondary structure at 37°C. This method can also be used for other organisms, especially for organisms still without characterized RNA secondary structures.

With the development of high-throughput sequencing techniques over the past half-decade, methods for RNA structure probing have improved from single-gene analysis to whole-transcriptome interrogation. Therefore, we can produce a map of the ‘RNA structurome’ in a given organism to investigate the structural landscape of eukaryotic transcriptomes and highlight the relationship between some specific RNA structures and functions (Kwok et al., 2015). Chemical modification or RNase digestion are two usually powerful methods that have been used most extensively to map RNA structure *in vivo* and *in vitro* (Lucks et al., 2011; Mortimer et al., 2014; Wildauer et al., 2014; Lee et al., 2017). Recently, a new chemoaffinity structure probing methodology, icSHAPE (*in vivo* click selective 2-hydroxyl acylation and profiling experiment), using a novel bifunctional chemical probe, NAI-N_3_, for *in vivo* RNA structure profiling in the genome has become a well-established tool for the analysis of RNA structure, and labelling each accessible single-stranded nucleotide provides a higher-resolution picture of the secondary structure of each transcript (Spitale et al., 2015).

Using the new chemoaffinity structure probing methodology icSHAPE, we generated a map of the ‘RNA structurome’ in *Plasmodium*, investigated the structural landscape of transcriptomes and highlighted the relationship between some specific RNA structures and their functions. Comparison of the static snapshots of our RNA structurome analysis in two developmental stages showed that the RNA structurome at two developmental stages possessed dynamic developmental responsive reorganization, long-range structures and higher-order architectures across the *Plasmodium* transcriptome. There was only 329 valid structural profiles for transcripts were obtained from *in vitro* 37°C treated polyA-selected RNA trophozoite stage libraries for the following reasons. First, the structural profiles that we define to valid structural profiles for transcripts are based on the transcripts which the reverse transcription stop coverage no less than 2 and background base density higher than 200. This is stricter than previously research. Second, the main reasons of less structural profiles from protein coding RNAs at the trophozoite stage compared to the ring stage is the low number of raw reads in trophozoite stage. Lower number of raw reads, less structural profiles. For the current study, we would like to focus on resolution and genome-wide RNA secondary structural in *P. falciparum* and the connection between RNA secondary structure and mRNA abundance during the complex biological program of *P. falciparum*. We therefore have not performed additional *in vitro* icSHAPE experiments (trophozoite stage) even with low coverage. *In vitro* structural profiles from protein coding RNAs at the trophozoite stage is for further investigation.

Although there is significant global correspondence between our icSHAPE-modified RNA structures and known structures, there are still some differences between icSHAPE and known structures; one reason for the differences is the noise in our approach, and the other reason is the known inaccuracies of folding algorithms (Kertesz, 2010). Therefore, these NAI-N_3_-independent stops occurred at known endogenously modified rRNA nucleotides; thus, we conclude that icSHAPE can successfully and accurately map chemically modified nucleotides on large RNAs on a genome-wide basis from *Plasmodium* parasites. Due to some nucleotides that were crowded *in vivo* and/or protein binding or experimental limitations, they were not chemically reactive, and no complete coverage was provided in our single experiment for this long transcript. Our partial but accurate RNA secondary structure can provide important information at some vital position of each transcript. Our icSHAPE results are strongly correlated with the results from our capillary electrophoresis-based probing method, and it can be used for accurate mapping of the RNA secondary structure. Our *in vivo* icSHAPE data are in excellent agreement with known RNA structures.

Although icSHAPE RNA secondary data were obtained from two stages of the *Plasmodium* and many genes expressed in other asexual developmental stages or mosquito/liver stages could not be obtained here, this study still provides important information on RNA secondary structure and the changes in structures between the two stages. Our study may have had several limitations. The depth was not enough to cover all of the whole transcripts, and larger studies with longer follow-up are needed to obtain RNA secondary structure information from the very low-level transcripts. Recently, a method named hiCLIP (Sugimoto et al., 2015) (RNA hybrid and individual nucleotide resolution ultraviolet crosslinking and immunoprecipitation), uses crosslinking and proximity ligation to determine a special RNA structures bound by special double-stranded RNA-binding proteins. This method may be a good choice for the determination of low-level transcripts or transcripts with complex RNA duplexes.

At present, researchers have found that mutations in many RNA helicases may lead to cancers (Fuller-Pace, 2013; Sullenger and Nair, 2016). Therefore, the application of the RNA secondary structure determination methods to these diverse problems and our parasites field will help us to identify some basic mechanisms of gene expression and potential therapeutic opportunities for treating diseases and parasites. Our findings also open avenues for the influence of specific RNA structural features on gene expression. It has yielded insights into the regulatory roles of RNA secondary structure in many organisms and cellular conditions (Wan et al., 2011; Strobel et al., 2018; Aw et al., 2020).

The development of *Plasmodium* parasites requires two hosts and completes 11 different stages. Therefore, there is a quick efficient response to various environmental changes. In the future, *in vivo* RNA structure studies under different stress conditions, such as temperature (Fang and McCutchan, 2002), the host body of nutrient supply (Mancio-Silva et al., 2017), amino acid (Babbitt et al., 2012) and glucose concentration in the blood (Fang et al., 2004), could provide clues about the structural characteristics of these dynamic mRNAs and their regulatory roles (Kwok et al., 2015).

In summary, our study presents the first icSHAPE-enabled transcriptome-wide secondary structure map and adds to the accumulating evidence that there is a critical connection between RNA structure and the complex biological programme of *P. falciparum*. Our results showed that the average icSHAPE score over the CDS revealed a very clearly correlation with the mRNA abundance. This means that stage specific RNA structure also play an important role in the regulation of gene expression in *P. falciparum*.

## Data Availability Statement

The datasets presented in this study can be found in online repositories. The names of the repository/repositories and accession number(s) can be found in the National Center for Biotechnology Information (NCBI) Sequence Read Archive under the BioProject ID PRJNA625343.

## Author Contributions

YQ designed and performed the experiments, analysed data, prepared figures, writing, review, and editing original draft. YZ and GZ designed and performed the experiments, analysed data, and prepared figures. BC and MZ prepared figures and performed analysis. JL, TP, and JH review and editing. XW designed and supervised the study. All authors performed data quantification, discussed the results, and commented on the manuscript. All authors contributed to the article and approved the submitted version.

## Funding

This research was funded by Natural Science Foundation of China (Grant number: 81902087), Guangdong Natural Science Fund Project (Grant number: 2017A030310535), and China postdoctoral Science Foundation (Grant number: 2016M592474).

## Conflict of Interest

The authors declare that the research was conducted in the absence of any commercial or financial relationships that could be construed as a potential conflict of interest.

## Supplementary Material

Supplementary Figure 1 Determination of the conditions for single-hit kinetics Determination of the conditions for single-hit kinetics. Time course of NAI-N_3_ modification and capillary electrophoresis data accurately maps to known structures. Lysis parasites were NAI-N_3_ treated for different durations (5 min, 10min, 15 min and 30 min) with the final NAI-N_3_ concentration was 100 mM. The 18S A-type rRNA NAI-N_3_ modification read-out was assessed by capillary electrophoresis-based probing, which was done here near the 5’ end (200bp-250bp, the region between two orange peaks). “5 min”, “10min”, “15 min” and “30 min” show the parasite lysis were incubated with 100mM NAI-N_3_ for 5 min, 10min, 15 min and 30 min at 37°C water bath respectively. “Control” show the parasite lysis were incubated with DMSO for 30 min at 37°C water bath. The red-blue stripe below “15 min” indicate the double-(red) or single-stranded (blue) RNA in this region.

Supplementary Figure 2 Determination of fragmentation time by primer extension electropherograms Determination of fragmentation time by primer extension electropherograms. Appropriate sizes of fragments are need for efficient obtain modification sites *via* RT-PCR and sequencing. Short RNAs, ~100 nt, are the most strategies that have been optimized to achieve single-hit kinetics of chemical modification. “1 min”, “2min”, “3 min” and “4 min” show the total RNA were fragmented for 1 min, 2min, 3 min and 4 min at 70°C PCR machine respectively. “Control” show the total RNA were no fragmented. The orange pink is GeneScan 500LIZ size standard, provides 5 single-stranded labeled fragments of: 50, 75, 100, 139 and 150 nucleotides.

Supplementary Figure 3 Secondary structure of *Plasmodium falciparum* 18S small subunit ribosomal RNA (blood stage) *in vivo* NAI-N_3_ modification identified by icSHAPE.

Supplementary Figure 4 Overview of the icSHAPE approach in our experiment Overview of the icSHAPE approach used for temperature-responsive *in vivo* and *in vitro* RNA structurome of *Plasmodium falciparum* in our experiment. P. falciparum was cultured in human O+ erythrocytes at 5% hematocrit at 37°C. Cultures were synchronized twice at ring stage with 5% D-sorbitol treatments performed 8 hours apart. We performed to culture parasites at 48/72 (ring stage/trophozoite stage) hours after the first synchronization experiment. Then parasites were lysis by 1% saponin, following by RNA structure modification by NAI-N_3_ or extract total RNA from parasite directly. During NAI-N_3_ modification, we set a DMSO-treated negative control sample for that the DMSO sample can provides an ‘input’ sample. *In vitro* RNA structure experiments were done by treating the total RNA from DMSO-treated sample with NAI-N_3_. After library preparation and Illumina sequencing, the count of mapping reads from sequencing generated NAI-N_3_ *in vitro*, NAI-N_3_ *in vivo*, and DMSO-treated control profiles. Finally, visualization the RNA secondary structure at difference stages and find the relationship between RNA secondary structure and gene expression.
