# Supplementary figures and images for: In Vivo and In Vitro Genome-Wide Profiling of RNA Secondary Structures Reveals Key Regulatory Features in Plasmodium falciparum
Source: Front Cell Infect Microbiol. 2021 May 17;11:673966. doi: 10.3389/fcimb.2021.673966 (PMC8166286; doi:10.3389/fcimb.2021.673966)

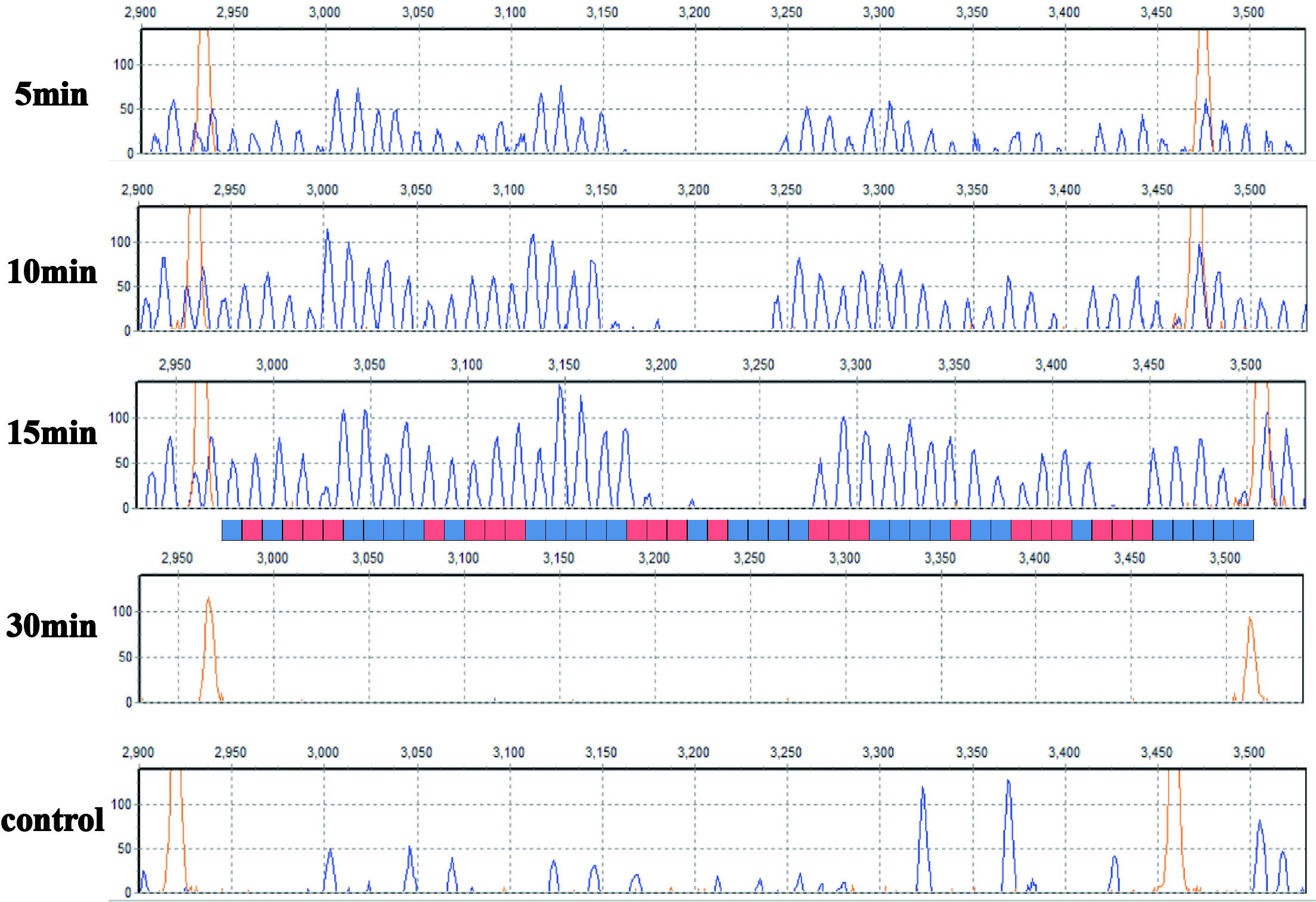

Supplement: Supplementary Figure 1 — Determination of the conditions for single-hit kinetics Determination of the conditions for single-hit kinetics. Time course of NAI-N3 modification and capillary electrophoresis data accurately maps to known structures. Lysis parasites were NAI-N3 treated for different durations (5 min, 10min, 15 min and 30 min) with the final NAI-N3 concentration was 100 mM. The 18S A-type rRNA NAI-N3 modification read-out was assessed by capillary electrophoresis-based probing, which was done here near the 5’ end (200bp-250bp, the region between two orange peaks). “5 min”, “10min”, “15 min” and “30 min” show the parasite lysis were incubated with 100mM NAI-N3 for 5 min, 10min, 15 min and 30 min at 37°C water bath respectively. “Control” show the parasite lysis were incubated with DMSO for 30 min at 37°C water bath. The red-blue stripe below “15 min” indicate the double-(red) or single-stranded (blue) RNA in this region. [file Image_1.jpeg]

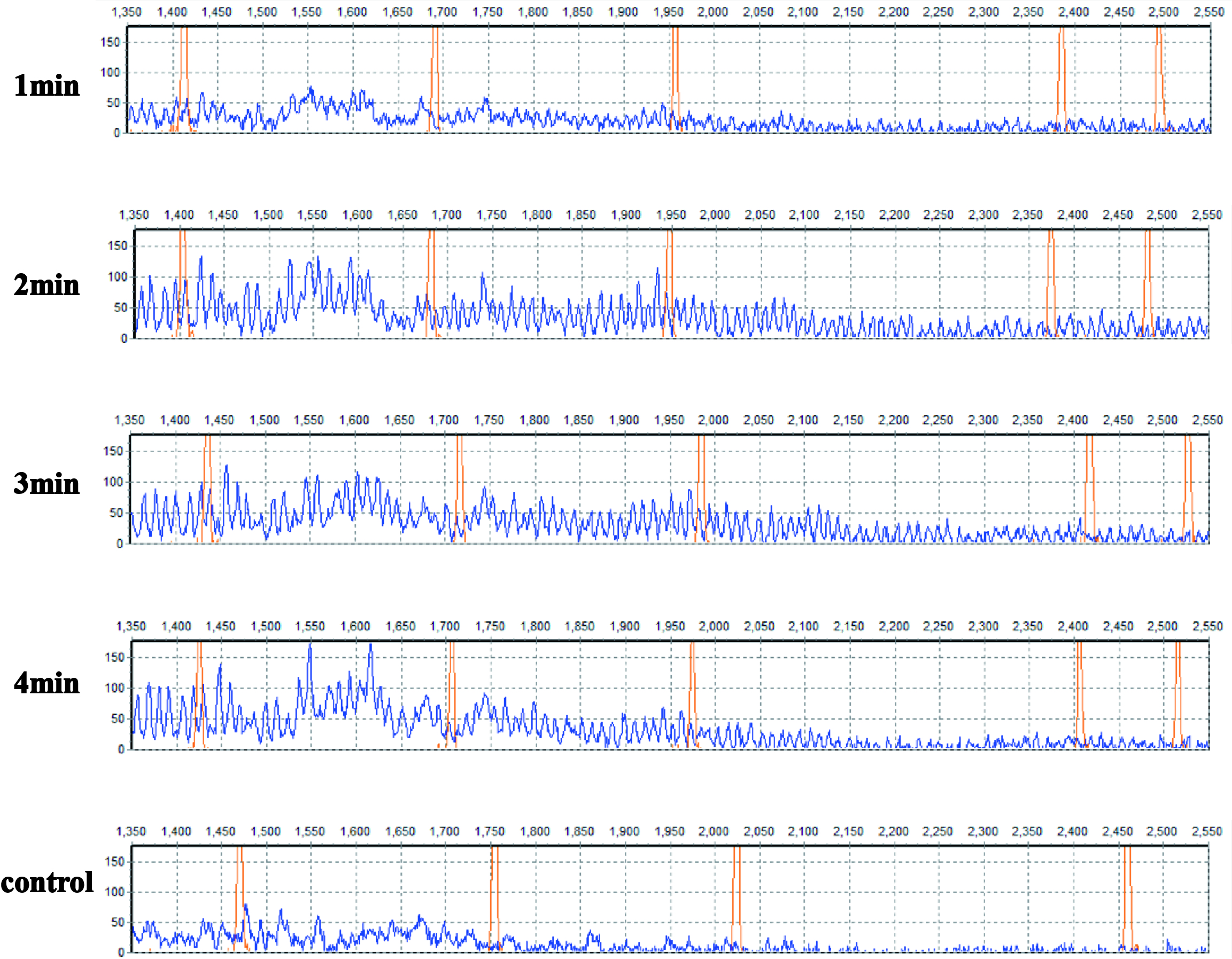

Supplement: Supplementary Figure 2 — Determination of fragmentation time by primer extension electropherograms Determination of fragmentation time by primer extension electropherograms. Appropriate sizes of fragments are need for efficient obtain modification sites via RT-PCR and sequencing. Short RNAs, ~100 nt, are the most strategies that have been optimized to achieve single-hit kinetics of chemical modification. “1 min”, “2min”, “3 min” and “4 min” show the total RNA were fragmented for 1 min, 2min, 3 min and 4 min at 70°C PCR machine respectively. “Control” show the total RNA were no fragmented. The orange pink is GeneScan 500LIZ size standard, provides 5 single-stranded labeled fragments of: 50, 75, 100, 139 and 150 nucleotides. [file Image_2.jpeg]

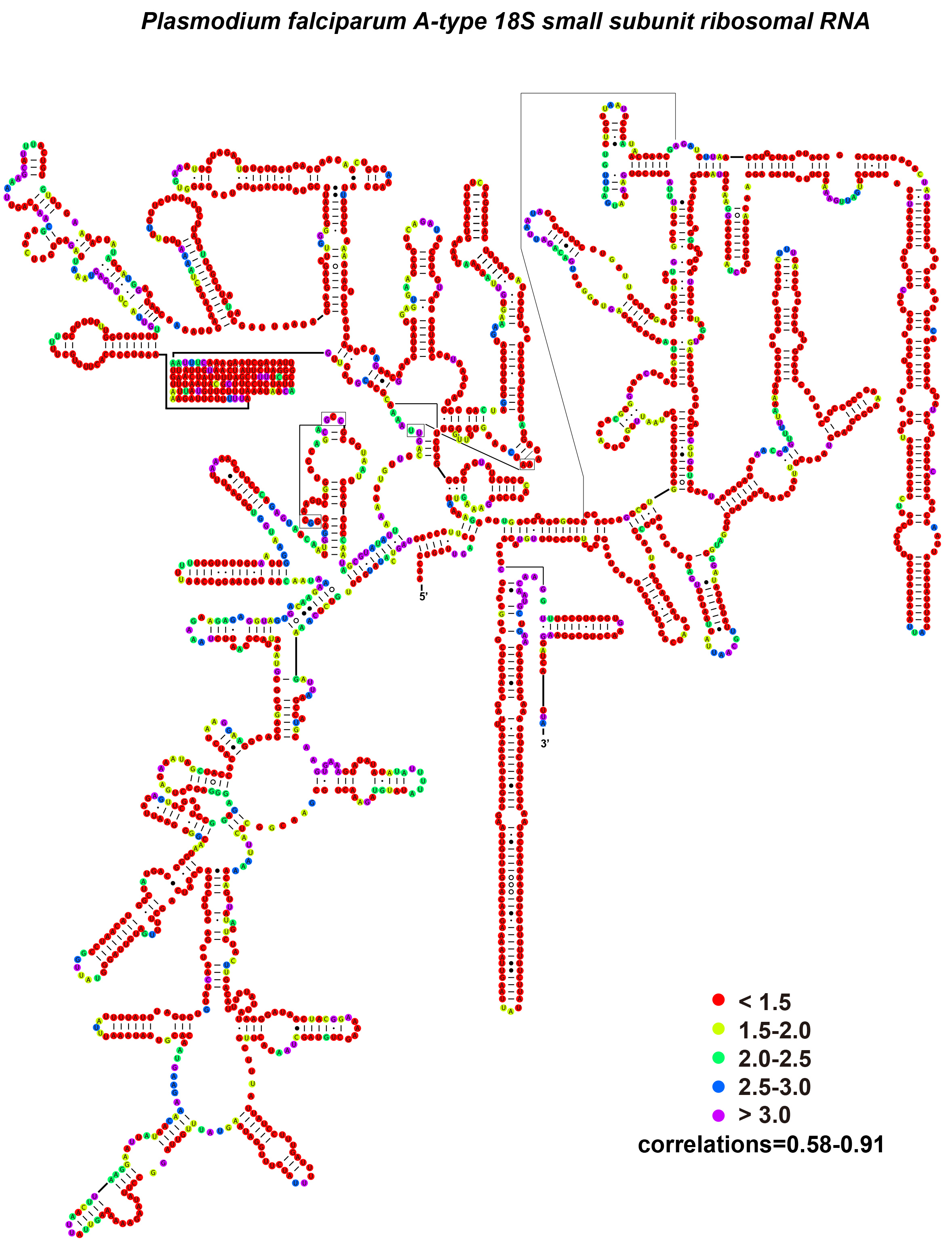

Supplement: Supplementary Figure 3 — Secondary structure of Plasmodium falciparum 18S small subunit ribosomal RNA (blood stage) in vivo NAI-N3 modification identified by icSHAPE. [file Image_3.jpeg]

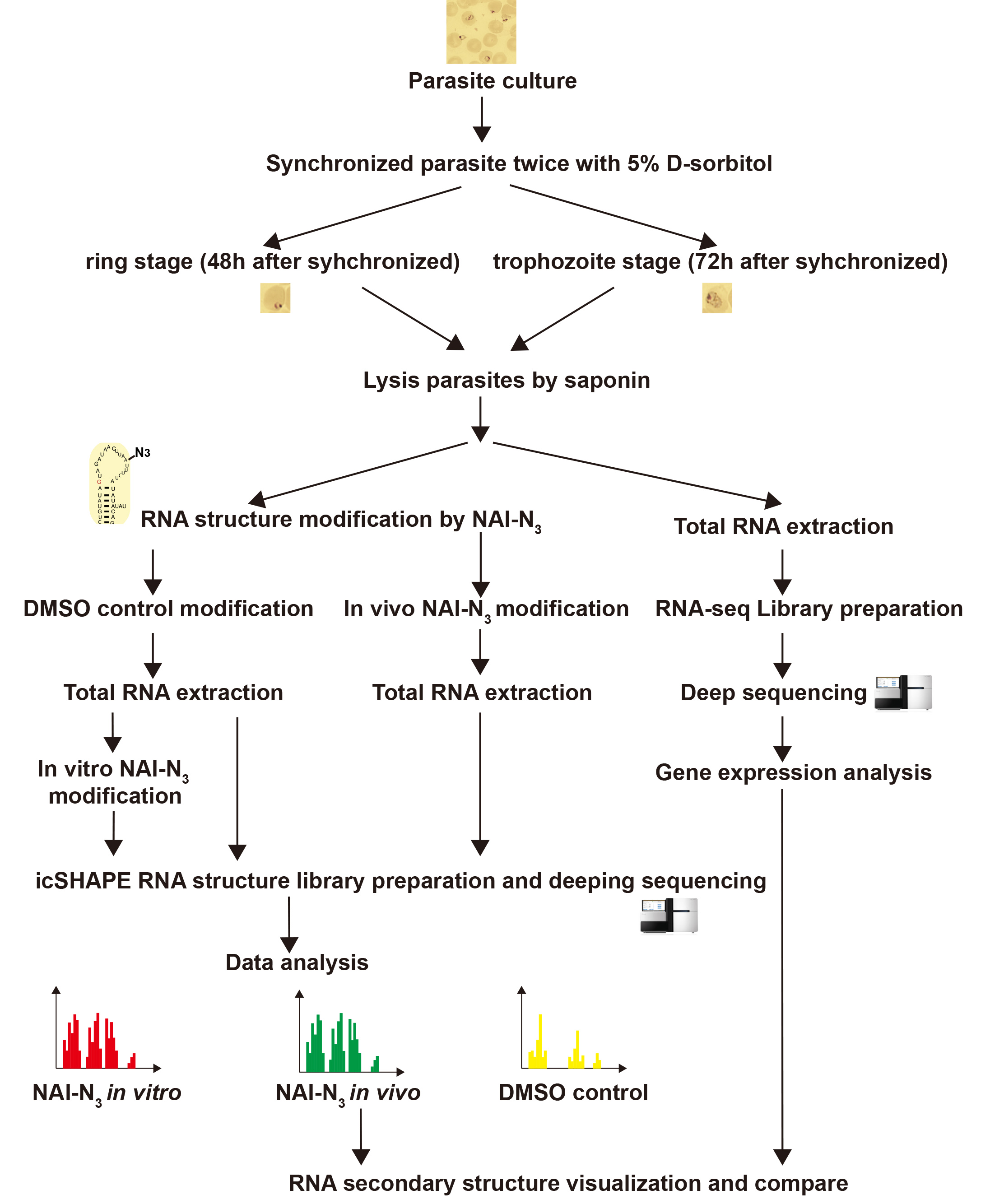

Supplement: Supplementary Figure 4 — Overview of the icSHAPE approach in our experiment Overview of the icSHAPE approach used for temperature-responsive in vivo and in vitro RNA structurome of Plasmodium falciparum in our experiment. P. falciparum was cultured in human O+ erythrocytes at 5% hematocrit at 37°C. Cultures were synchronized twice at ring stage with 5% D-sorbitol treatments performed 8 hours apart. We performed to culture parasites at 48/72 (ring stage/trophozoite stage) hours after the first synchronization experiment. Then parasites were lysis by 1% saponin, following by RNA structure modification by NAI-N3 or extract total RNA from parasite directly. During NAI-N3 modification, we set a DMSO-treated negative control sample for that the DMSO sample can provides an ‘input’ sample. In vitro RNA structure experiments were done by treating the total RNA from DMSO-treated sample with NAI-N3. After library preparation and Illumina sequencing, the count of mapping reads from sequencing generated NAI-N3 in vitro, NAI-N3 in vivo, and DMSO-treated control profiles. Finally, visualization the RNA secondary structure at difference stages and find the relationship between RNA secondary structure and gene expression. [file Image_4.jpeg]
